# Physicochemical and Optical Characterization of *Citrus aurantium* Derived Biochar for Solar Absorber Applications

**DOI:** 10.3390/ma14164756

**Published:** 2021-08-23

**Authors:** Nancy G. Gonzalez-Canche, Jose G. Carrillo, Beatriz Escobar-Morales, Iván Salgado-Tránsito, Neith Pacheco, Soledad Cecilia Pech-Cohuo, Manuel I. Peña-Cruz

**Affiliations:** 1 Centro de Investigaciones en Óptica, A.C. Unidad Aguascalientes, Prol. Constitución 607, Fracc. Reserva Loma Bonita, Aguascalientes 20200, Mexico; nancy.gonzalezcanche@gmail.com; 2 Centro de Investigación Científica de Yucatán, Unidad de Materiales, Calle 43 No. 130, Chuburná de Hidalgo, Mérida, Yucatán 97205, Mexico; jgcb@cicy.mx; 3 CONACYT-Centro de Investigación Científica de Yucatán, Carretera Sierra Papacal-Chuburná Puerto, Km 5, Mérida, Yucatán 97302, Mexico; beatriz.escobar@cicy.mx; 4 CONACYT, Centro de Investigaciones en Óptica, A.C. Unidad Aguascalientes, Prol. Constitución 607, Fracc. Reserva Loma Bonita, Aguascalientes 20200, Mexico; isalgadotr@cio.mx; 5 Centro de Investigación y Asistencia en Tecnología y Diseño del Estado de Jalisco CIATEJ, A.C. Subsede Sureste, Parque Científico Tecnológico de Yucatán, Km 5.5 Carretera Sierra Papacal-Chuburná Puerto, Mérida, Yucatán 97302, Mexico; npacheco@ciatej.mx (N.P.); spech_al@ciatej.edu.mx (S.C.P.-C.)

**Keywords:** agro-industrial waste, biochar, solar absorber

## Abstract

Agro-industrial waste valorization is an attractive approach that offers new alternatives to deal with shrinkage and residue problems. One of these approaches is the synthesis of advanced carbon materials. Current research has shown that citrus waste, mainly orange peel, can be a precursor for the synthesis of high-quality carbon materials for chemical adsorption and energy storage applications. A recent approach to the utilization of advanced carbon materials based on lignocellulosic biomass is their use in solar absorber coatings for solar-thermal applications. This study focused on the production of biochar from *Citrus aurantium* orange peel by a pyrolysis process at different temperatures. Biochars were characterized by SEM, elemental analysis, TGA-DSC, FTIR, DRX, Raman, and XPS spectroscopies. Optical properties such as diffuse reflectance in the UV−VIS−NIR region was also determined. Physical-chemical characterization revealed that the pyrolysis temperature had a negative effect in yield of biochars, whereas biochars with a higher carbon content, aromaticity, thermal stability, and structural order were produced as the temperature increased. Diffuse reflectance measurements revealed that it is possible to reduce the reflectance of the material by controlling its pyrolysis temperature, producing a material with physicochemical and optical properties that could be attractive for use as a pigment in solar absorber coatings.

## 1. Introduction

Agro-industrial and food waste are an alarming problem on the planet. The numbers are outstanding—almost 1 billion tons of edible food waste are thrown out each year worldwide and the total food supply chain is of several billions. This is particularly astounding when considering that the carbon value of these waste sources is comparable to those in all chemical and plastics used each year, but with the advantage that the former is renewable [1]. The appropriate food and agro-waste management for the production of value-added products could increase the efficiency of the food supply chain and reduce its associated cost, improving accessibility to food security. Furthermore, the use of agro-residues as a source of natural products and value-added chemicals could promote the necessary investments to reduce the negative socioeconomic and environmental impacts caused by food and agro waste; for this purpose, plenty of research efforts are necessary in the short term to guarantee suitable progress in this field [2]. In this sense, the valorization of this type of waste is an attractive approach that could offer useful alternatives through the production of value-added chemicals, fuels, and advanced carbon materials [1,3]. In particular, in Mexico, an average of 4.2 million metric ton of citrus is produced per year, from which 90% is dedicated to fresh consumption and 10% is used in the juice and concentrates industry, generating a high amount of waste, where 40−60% of citrus becomes waste, including peels, seeds, and membranes [4,5]. 

Literature shows that the extraction of useful chemical compounds from citrus waste is an attractive approach to its valorization. An example is limonene and terpineol extraction, which are valuable chemicals as fragrances, solvents, and chemical intermediates [1]. Another example is the extraction of compounds such as pectin, polyphenolic, and flavonoid compounds from orange peel, mainly of the *Citrus sinensis* variety, for nutraceutical and pharmaceutical applications [3,6,7].

On the other hand, the literature shows that high quality carbon materials can be produced from this low valued precursor, which could offer an effective method for conversion into high valued products [3]. There are different studies that focus on the preparation and characterization of activated carbon from orange peel for different applications related to the adsorption properties, such as the remotion of metals, dyes, herbicides, and other contaminants [8,9,10,11], whereas other studies are focused on energy storage applications like supercapacitors [12]. There are also reports related to its integral valorization of orange peel by pectin extraction and its subsequent conversion to activated carbon [3].

Among other varieties of citrus fruits, sour orange (*Citrus aurantium*) is less used for fresh consumption due to its sourness; instead, sour orange juice is often used as an ingredient in meat marinades or salad dressings. For the *Citrus sinensis* variety, current research has shown that sour orange peel (SOP) can be used as a source of added value compounds such as essential oils, pectin, phenolic compounds, and flavonoids, as well as flavoring and acidifying agents for food [5,7,13,14,15]. 

A different approach to the conversion of SOP into a high value product is the preparation of carbon-based materials for solar energy applications. For example, the literature shows that carbon materials, such as reduced graphene oxide, can be precursors for the synthesis of functional covalent nanocarbon hybrids with metal-free donor-ℼ-acceptors that could be applied as photosensitizing agents in dye-sensitized solar cells [16]. The literature also shows the potential of carbon-based materials as solar absorbers for solar thermal applications. Studies performed by López-Sosa et al. showed that carbon-based materials can be used in solar absorber coatings, where the coating involved the use of soot produced by the combustion of forest resources in Patsari firewood-saving stoves, and the presence of pseudo-amorphous carbon exhibited a solar absorption capacity due to sp^2^−sp^3^ bonds present in this kind of carbon source [17]. However, there are a lack of studies related to the synthesis and characterization of carbon-based materials from agro-industrial waste, such as *Citrus aurantium* peel for the obtention of sustainable solar absorber materials, and the effect of synthesis conditions, such as temperature, on the physicochemical and optical properties, which could offer an innovative approach to the use of this kind of residue as pigments in the formulation of solar absorber coatings, contributing to reducing the negative socioeconomic and environmental impacts caused by citrus agro-waste. 

The objective of this study is to investigate the production of carbonaceous materials through the pyrolysis of *Citrus aurantium* orange peel and to evaluate the resulting characteristics (elemental composition, TGA-DSC, FTIR, XRD, Raman, and XPS spectroscopy) and optical properties (diffuse reflectance in UV−VIS−NIR) resulting from using different temperatures in the pyrolysis process, to identify the best characteristics for possible use in solar absorber coatings.

## 2. Materials and Methods

### 2.1. Raw Material

Orange fruits (*Citrus aurantium*) were purchased from a local market in Yucatán, México. In order to obtain a raw material that can be used in the extraction of value-added chemicals and in the synthesis of biochars, sour oranges were peeled (SOP), and the fresh peels were shredded and dried in a steam dehydrator (Jersa 148-09, Ciudad de México, México) with air circulation at 45 °C for 24 h. The drying conditions were fixed to favor the extraction of significant amounts of value-added chemicals such as polyphenols from SOP [18]. Subsequently, the sour orange peels were finely ground (Pulvex 200 grinder, Ciudad de México, Mexico) and passed through a < 0.500 mm metal sieve. The raw powder material obtained was called sour orange peel (SOPRAW).

### 2.2. Pyrolysis Experiments

Pyrolysis experiments were performed in a tubular furnace OTF-1200 X MTI Corporation in a nitrogen atmosphere. Before the pyrolysis experiments, the SOPRAW samples were dried for a second time in a convection oven at 70 °C for 24 h to remove any residual moisture content present after obtaining the powder raw material. Samples of dried SOPRAW weighing 2.00 g were placed in the tubular furnace and then heated at a heating rate of 10 °C/min, from room temperature to 400 °C, 600 °C, and 800 °C over 1 h with a nitrogen inert gas flow of 50 mL/min. 

The carbonization yield (CY) was calculated using the mass remaining from the carbonization process (g):

CY (%) = (m_SOPCBN_/m_SOPRAW_) × 100 (1)

where m_SOPRAW_ is the SOPRAW dried mass used in the preparation of carbon materials in the pyrolysis experiments and m_SOPCBN_ is the final amount of carbon after the carbonization process.

### 2.3. Physicochemical Characterization

#### 2.3.1. Morphology

The morphology of SOPRAW, before pyrolysis, and the biochars obtained from the pyrolysis process at 400, 600, and 800 °C (SOP400, SOP600, and SOP800, respectively) were examined using scanning electron microscopy (SEM) on a low vacuum scanning electron microscope JEOL JSM 6360 (JEOL, Tokyo, Japan).

#### 2.3.2. Elemental Analysis

Elemental analysis was carried out using an Organic Elemental Analyzer Flash 2000 CHNS-O (Thermo Scientific, Milan, Italy) coupled with EAGER experience software, version 1.2. The elemental composition was determined in triplicate for one representative sample of each condition, and the oxygen content was calculated using the difference method. Before carrying out the elemental analysis, the preparation of samples of SOPRAW and biochars involved drying at 105 °C for 24 h to reduce the possible effects of moisture content, in this way obtaining the elemental composition on a dry basis.

#### 2.3.3. Fourier Transform Infrared (FTIR) Spectroscopy

FTIR spectroscopy was carried out on a Bruker Tensor II spectrometer (Bruker, Billerica, MA, USA). The detection and collection of the spectrum range was 4000–500 cm^−1^, with a spectral resolution of 4 cm^−1^. A number of 32 scans were recorded for each sample using the attenuated total reflectance (ATR) technique.

#### 2.3.4. Thermal Analysis (TGA-DSC)

Thermogravimetric analysis and differential thermal analysis were performed in a TGA-DSC simultaneous analyzer NETZSCH STA 449 F5((NETZSCH, Selb, Germany). Sample mass was kept at 10 mg. The samples were heated from room temperature up to 900 °C at a constant heating rate of 10 °C/min using a He atmosphere and a flow rate of 20 mL/min.

#### 2.3.5. X-ray Diffraction (XRD)

X-ray diffraction (XRD) was employed to compare the differences of the XRD patterns of the raw material and biochars. XRD patterns of the samples were determined using a Bruker D2 Phaser apparatus (Bruker, Karlsruhe, Germany) with a step size of 0.0101 ϴ degrees and 4.75 step per second, in a range from 9.99 to 99.99 2ϴ degrees.

#### 2.3.6. Raman Spectroscopy

Once the carbon was obtained, the graphitic character was evaluated using Raman spectroscopy in a Renishaw inVia Raman microscope (Renishaw, Gloucestershire, UK) using a 532 nm laser.

#### 2.3.7. X-ray Photoelectron (XPS) Spectroscopy

The chemical composition of the materials surface was characterized through X-ray photoelectron spectroscopy (XPS) using Thermo Scientific spectrophotometer (Mod K-Alpha) (Thermo Scientific, East Grinstead, UK) with an Al-Kα source al 12 kV. Spectral calibration was done using C 1s at 284.5 eV as reference.

### 2.4. Optical Characterization

In this procedure, the spectra of diffuse reflectance of SOPRAW and biochars were measured (0.3–2.5 µm) using a Cary 5000 UV−VIS−NIR spectrophotometer (Agilent Technologies, Santa Clara, CA, USA) equipped with an integration sphere.

## 3. Results and Discussion

### 3.1. Carbonization Yield

The effect of the pyrolysis temperature on the biochar carbonization yield (CY) is shown in Table 1, where it is observed that yield decreased with the increase of pyrolysis temperature; the highest yield was produced at 400 °C, while the lowest was produced at 800 °C, observing a further thermal decomposition of the SOP constituents. Table 1 also shows a yield decrease of 7.52% from 400 °C to 600 °C and a moderate decrease of 1.69% from 600 °C to 800 °C. As a lignocellulosic material, sour orange peel can be composed of three main building blocks, namely hemicellulose, cellulose, and lignin, as well as some extractives [19], it is expected that temperature increase favors the decomposition of these constituents as well as the release of gases and volatiles caused by this decomposition [20]. A decrease in yield of 7.52% from 400 °C to 600 °C suggests that most of the lignocellulosic material was decomposed, while a decrease of 1.69% from 600 °C to 800 °C, which is much smaller than the previous amount, suggests the formation of more stable biochar with a much lower material loss. This negative effect of temperature on the carbonization yield is consistent with previous studies, such as that reported by Chaves Fernandes et al., where a negative correlation between the pyrolysis temperature and the Eucalyptus biochar yield was found, mainly in the range of 450–650 °C [20]. Similar behaviors have been shown in other carbonaceous materials, which presented this reduction of yield at higher temperatures until they became lower and constant. Li et al. reported the same negative effect on the yield of the biochars obtained from different feedstocks, where a decrease in yield was observed, whereas an increase in bio-oil and syn gas yields was shown with an increase in temperature [21]. On the other hand, the decrease of yield was moderate as the temperature increased, which is attributed to a major carbonization is completed at temperatures in the range of 600–800 °C [20,21].

### 3.2. Morphology

**Figure 1:**
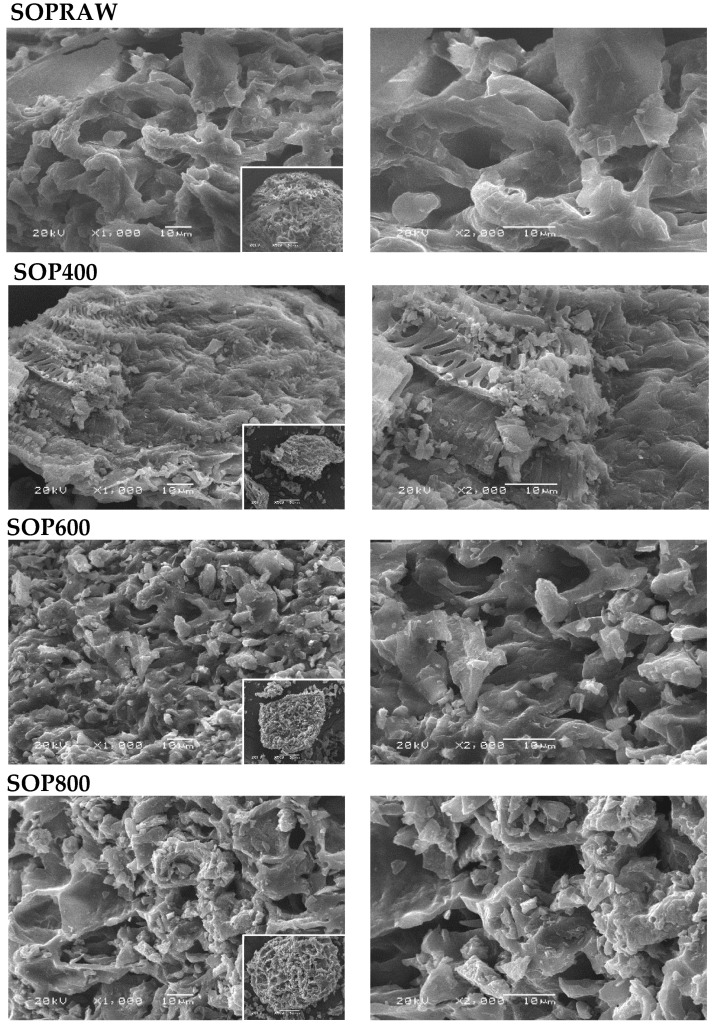
SEM images of SOPRAW, SOP400, SOP600, and SOP800.

A structural image such as SEM plays an important role in understanding the main changes on the surface of the raw material and biochar produced. The morphological features of SOPRAW and biochars SOP400, SOP600, and SOP800 are shown in Figure 1. It can be observed that the morphology of the raw material SOPRAW shows a channel-like morphology with cavities, but after the pyrolysis process at different temperatures, the morphology changes and the decomposition is evident. The micrography of SOP400 shows a morphology that suggests a partial decomposition of SOPRAW, with remnants of these channels; in addition to this, the presence of small and agglomerated particles is observed. The micrography of SOP600 shows a morphology with more cavities and a higher number of small particles in comparison with SOP400. The same effect is shown in SOP800 in comparison with the other biochars, where the particles have a structure of more cavities and small particles that are agglomerated, which suggests that a higher degree of decomposition is achieved by the increase in pyrolysis temperature. Increasing the pyrolysis temperature results in a growing proportion of biochar with smaller size distribution, as reported by Kim et al. [22].

In addition to this, it is interesting that through the pyrolysis process, it is possible to achieve a morphology with cavities, which could result attractive for photothermal materials. The literature shows that structures with pores and cavities are attractive morphologies in the synthesis of photothermal carbon-based materials, due to their contribution of improving light absorption and minimizing light reflection in the solar spectrum [23].

### 3.3. Elemental Analysis

Elemental analysis data can be related to the biochar stability [20]. As N and S are minor elements of biochar, the unsaturation or aromaticity may be assessed by the C, H, and O compositions and H/C and/or O/C ratios. The elemental composition of SOPRAW and biochars are summarized in Table 2, the moisture content of SOPRAW, determined by the gravimetric method according to the AOAC official method [24], was 9.328 ± 1.198 wt.%. As shown in Table 2, an increment in C content was observed as the pyrolysis temperature increased, whereas an inverse trend was observed for the H and O content. On the other hand, N and S were not significantly present in the samples. It is known that pyrolysis favors dehydration and deoxygenation, which promotes the elimination of H and O over C, resulting in the accumulation of C in solid residue biochar [25]. In this sense, the increase in C content and the decrease in H and O indicate that the loss of functional groups associated with SOP constituents, such as carboxyl, hydroxyl in hemicellulose, cellulose, and lignin of lignocellulosic materials, which indicates that as the pyrolysis temperature become higher, further thermal decomposition in SOPRAW is produced. On another hand, the O content can have an important effect in the biochar surface chemistry behavior, due to its close relationship with the number and composition of the substituted functional groups, which can constitute an important agent for the degradation potential [25].

Table 2 also shows the aromaticity (H/C) and the hydrophilicity (O/C) index for SOPRAW and biochars. The aromaticity index (H/C) may be used to assess the degree of thermochemical alteration that produces fused aromatic ring structures in the obtained material; then, the lower H/C means higher fused aromatic ring structures and higher stability [25]. This trend is observed in the produced biochars, which suggests that their aromaticity increases with the increase in pyrolysis temperature. The hydrophilicity index (O/C) also showed a decrease with the increase of temperature; this index is related to biochar stability, because the O content can lead degradation reactions, so the lower the O/C ratio, the higher the stability in carbon fraction [20,26,27]. In particular, the O/C ratio exhibited by SOP600 and SOP 800 corresponds to materials that can be considered black carbon. Conversely, the O/C ratio exhibited by SOP400 corresponds to those exhibited by the biomass, which suggests that a partial thermal decomposition of the raw material is achieved at 400 °C [28].

### 3.4. Fourier Transform Infrared (FTIR) Spectrocopy

**Figure 2:**
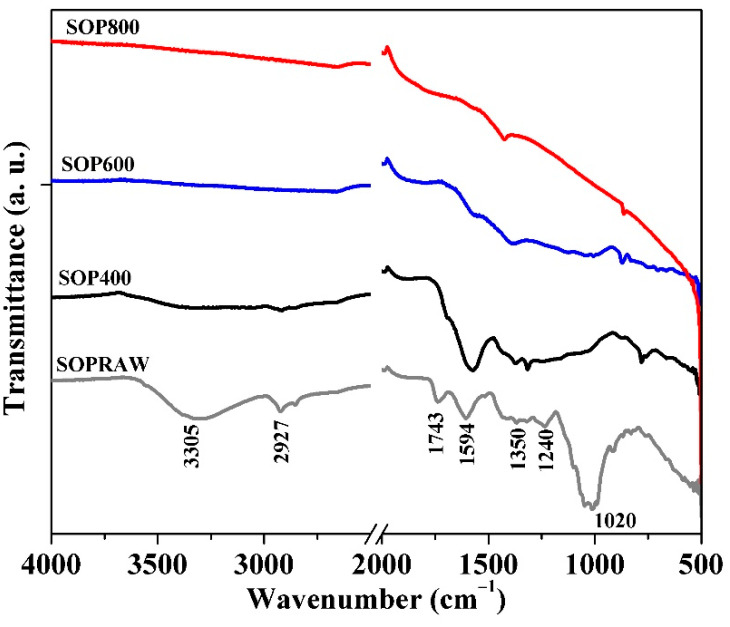
FTIR spectra of SOPRAW and SOP400, SOP600, and SOP800.

Figure 2 shows the FTIR spectra of SOPRAW, SOP400, SOP600, and SOP800, where differences attributed to the thermal decomposition of components in pyrolysis process of a lignocellulosic material as SOPRAW can be appreciated. Orange peel can be composed of three main building blocks, namely hemicellulose, cellulose, and lignin; other components also comprise some extractives, therefore, SOPRAW exhibits a typical spectra corresponding to a lignocellulosic material [9,19,29]. A broad absorption peak in the 3500–3000 cm^−1^ in SOPRAW corresponds to the O-H stretching vibration of cellulose, pectin, hemicellulose, lignin, and residual water in sour orange peel [30,31]; however, in SOP400, the intensity of this peak decreases and for SOP600 and SOP800 it is not present, because of the denaturing property of carbonization in the pyrolysis process that promotes components disintegration [30]. A similar effect is shown for the peak at 2927 cm^−1^ for SOPRAW, for which its intensity is much lower in SOP400 and it is not exhibited by SOP600 and SOP800. This peak is related to the aliphatic saturated C-H stretching vibration of the hemicellulose, cellulose, and lignin present in the precursor material [3]. Another peak is found at 1743 cm^−1^, which indicates the carbonyl (C=O) stretching vibration of the carboxyl groups of pectin, hemicellulose, cellulose, and lignin, which are present in SOPRAW only due to the dissociation of the carboxylic groups in pyrolysis process. The next peak at 1600 cm^−1^ is attributed to C=C stretching of aromatic rings in lignin, which is shown by SOPRAW and SOP400, and decreases significantly in SOP600 and SOP800. Peaks at 1400–1300 cm^−1^ could be attributed to aliphatic and aromatic groups in the plane methyl, methylene, and methoxy groups, while the peak at 1230 cm^−1^ is related to the aliphatic chains (-CH_2_ and -CH_3_) and methoxy groups (O-CH_3_) from the lignocellulosic material. Finally, the peak at 1000 cm^−1^ can be assigned to the C-O stretching vibration of alcohols and ester groups [3,20,32,33]. 

### 3.5. Thermal Analysis (TGA-DSC)

**Figure 3:**
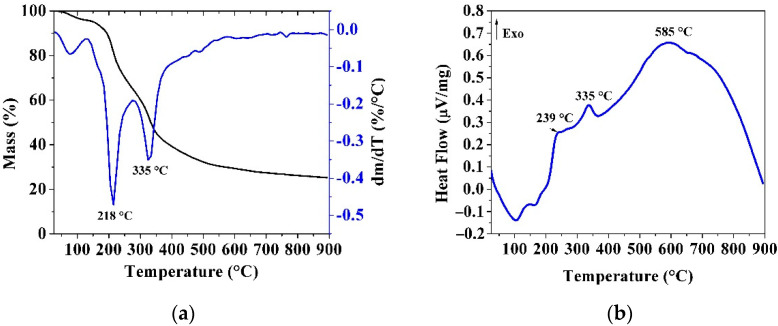
(**a**) TG-DTG curves; (**b**) DSC curves of SOPRAW.

The thermal profiles of SOPRAW obtained from the simultaneous TGA-DSC analyzer are shown in Figure 3. Figure 3a shows the TG-DTG curves, whereas Figure 3b shows the DSC curve. 

The TG-DTG curves show at least three different mass loss events up to 900 °C. The polymeric nature of the lignocellulosic materials is complex, and at least two mechanisms coexist during the pyrolysis process, a situation that may contribute to the observed overlap in the DTG curves [34,35]. Figure 3a shows a first mass loss (from 25 to 150 °C), related to moisture loss and extractives of the sour orange peel. The following mass losses can be associated with the biomass thermal degradation of its three main components hemicellulose, cellulose, and lignin, observed in the FTIR analysis. A second mass loss (from 150 to 270 °C) is characterized by a peak at 218 °C and an exothermic peak at 239 °C in the DSC curve in Figure 3b. This mass loss is attributed to the thermal degradation of hemicellulose, according to the reported results by Zapata et al. [35]. The third mass loss (270 to 380 °C) with an exothermic peak at 335 °C is related to the thermal degradation of cellulose, which is consistent with results reported by Monteiro Santos et al., due to the rigid structure of cellulose exhibiting a higher thermal stability than hemicellulose [9]. Lignin is an amorphous cross-linked resin with no exact structure, and its degradation occurs between 280 and 500 °C and is hindered by the decomposition of hemicellulose and cellulose, except at high temperature [34,35,36]. So, lignin in lignocellulosic materials is the last organic constituent to be decomposed to produce biochar. Studies have revealed that lignin decomposition can occur without observable peaks in the DTG curve [37], however this decomposition is observed in the DSC curve as an exothermic peak at 585 °C.

**Figure 4:**
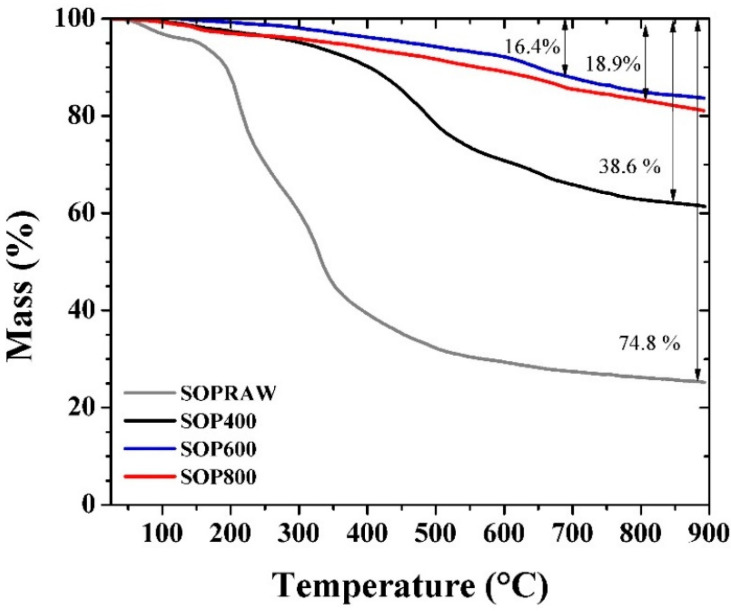
TG curves of the SOPRAW and biochars.

Figure 4 shows TG curves of SOPRAW and biochars, where SOPRAW shows a 75% approximate mass loss, whereas SOP400 shows a mass loss of 39%, which suggests that an incomplete decomposition is obtained, according to the DT-DTG and DSC analyses of SOPRAW, as a complete decomposition of lignin is achieved at higher temperatures. On the other hand, SOP600 and SOP800 show a lower loss of mass than those exhibited by SOPRAW and SOP400, and a decomposition behavior that becomes much lower as the temperature increases, suggesting that both biochars have a better thermal stability than SOP400. The thermal behavior of SOP600 and SOP800 are consistent with the elemental analysis and O/C ratio related to biochar stability, and are the converse of the O/C ratio exhibited by SOP400, corresponding to a biomass interval, which suggest that a partial decomposition of SOPRAW is achieved and secondary decomposition reactions may be developed during the TGA analysis.

In addition to this, a high thermal stability is an important property for a solar absorber material design. Typically, the low−medium temperature solar thermal devices achieve temperatures in the range of 60–400 °C [38], so the solar absorber surface requires materials with a thermal stability, according to this temperature range. As it can be observed, the thermal behavior of SOP600 and SOP800 shows that at 400 °C, only 5% of mass loss is approximately exhibited by the biochars, which suggests that these biochars could exhibit a good thermal stability for this range of temperature.

### 3.6. X-ray Diffraction (XRD) Analysis

**Figure 5:**
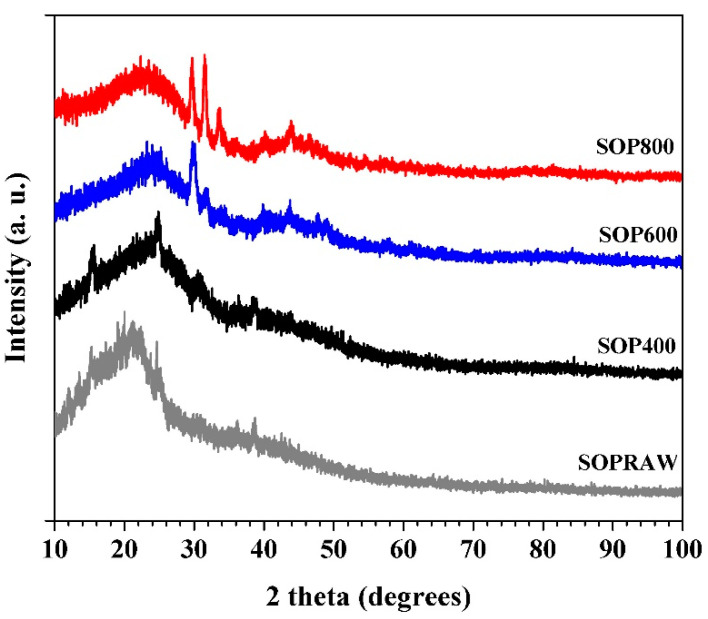
XRD patterns of SOPRAW and biochars SOP400, SOP600, and SOP800.

The crystallinity character of the SOPRAW and biochar samples was determined using XRD and their patterns are shown in Figure 5. The XRD patterns of SOPRAW shows that it has an amorphous structure, and the hump exhibited at 2theta around of 22° is related to the weak crystalline nature of the cellulose in SOPRAW, which implies the amorphous nature expected for the biochars [30,37,39]. The XRD patterns of the biochars show broad peaks located at 24° and 44°, these peaks are related to 002 and 100 planes of graphitic like structures, respectively, indicating the presence of amorphous carbon as well as a certain degree of graphitization. As it has been observed, displacement to higher diffraction angles from 22° to 24° is produced, due to the carbonization process of SOPRAW [40,41,42]. XRD patterns also show the effect of temperature in the pyrolysis process where the peak at 44° is more evident in SOP800, which suggests that a higher temperature in the pyrolysis process produces a higher degree of carbonization, as was observed in the FTIR analysis and elemental analysis [43]. Finally, a sharp peak at 29.5° in SOP600 and SOP800 was observed—this peak can be related to the presence of inorganic components such SiO_2_ and CaCO_3_, which suggests that with the increase of pyrolysis temperature, it produced a release of ash, for example, ashes contain alkali salts such as calcium carbonate [44,45].

### 3.7. Raman Spectroscopy

**Figure 6:**
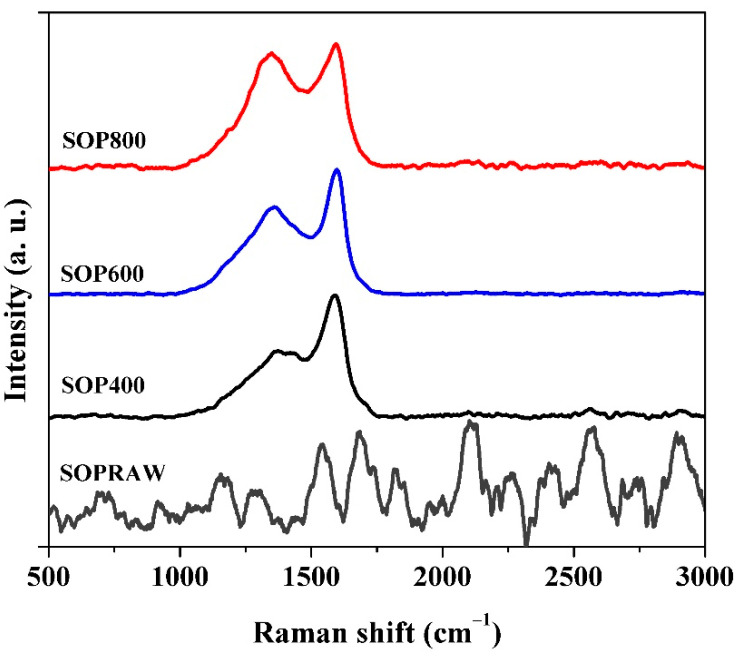
Raman spectra SOPRAW and biochars SOP400, SOP600, and SOP800.

Raman spectra of the SOPRAW and biochars obtained are shown in Figure 6. SOPRAW does not show any characteristic band, while SOP400, SOP600, and SOP800 show two broad and overlapping peaks, with a maximum intensity at 1363 cm^-1^ and 1590 cm^-1^, which correspond to the D and G bands of the graphitic-like structures, respectively [46]. The D band is related to sp^3^ type defective sites in the graphitic plane and disorder, while the G band results from the vibrational mode of the graphite crystalline planes [10,47]. 

**Figure 7:**
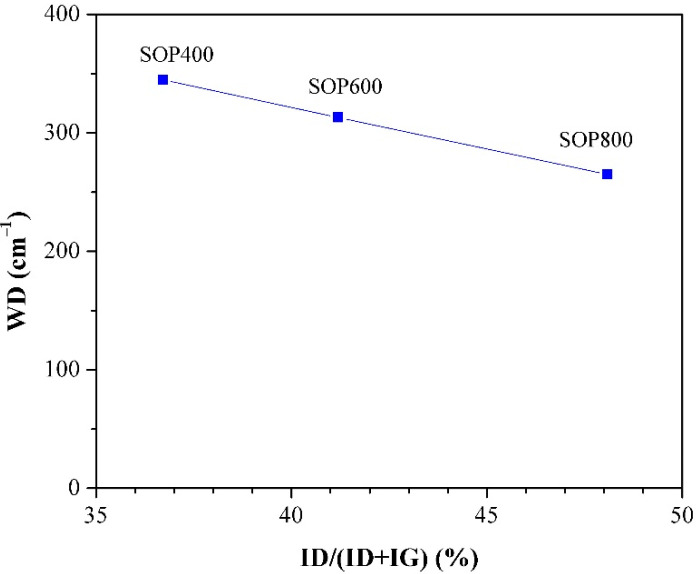
Raman evolution of D band width vs disorder parameter.

Structural parameters such as the D and G bands’ intensity ratios I_D_/I_G_ and I_D_/(I_D_ + I_G_), as well as the D band width (W_D_), are indicators of the biochar structure. Table 3 shows the (I_D_/I_G_) ratio and the disorder parameter I_D_/(I_D_ + I_G_), and Figure 7 shows the variation of the D band width (W_D_) versus the disorder parameter [48]. From Table 3, it can be observed that both increase with the pyrolysis temperature. Firstly, the I_D_/I_G_ ratio notable increased by approximately 60% from 400 to 800 °C, which suggests that there is a higher proportion of condensed aromatic structures with defects [32]. On the other hand, the increase of the disorder parameter with the pyrolysis temperature seems to indicate a decrease in the sp^2^ domain; however, the literature shows that temperature can favor the structural order in biochars [49]. The above is consistent with the previous XRD patterns, where peaks at 24 ° and 44° are better defined as the temperature increases, and then a complementary parameter, such as W_D_, can provide a more suitable comparison of the level or order of materials. The literature shows that low values of W_D_ can be related to biochars with a high level of order [48]. In this sense, the plot of W_D_ indicates that SOP800 is the most ordered biochar, whereas SOP400 is the least; therefore, an increasing level of order can be achieved through the increase in pyrolysis temperature, which is consistent with previous studies performed in lignocellulosic materials [32,48].

As can be observed, the biochars exhibit amorphous characteristics composed by a mixture of sp^2^ and sp^3^ bonds, where the increase in the pyrolysis temperatures favors the increase of level or order, and then the sp^2^ domain. In this sense, these characteristics are important in organic light absorber materials, whose main mechanism of photoconversion is the thermal vibration of molecules [23]. The literature shows that sp^2^−sp^3^ carbon allotropes have higher light absorption coefficients in comparison with known light absorbers, because their photothermal effect is associated with the sp^2^ domain of graphitic structures, which allows the absorption of light in the solar spectrum [23,50,51], which suggests that produced biochars could exhibit important differences in their optical properties according to the degree of sp^2^ domain and then the level of order.

### 3.8. X-ray Photoelectron (XPS) Spectroscopy

**Figure 8:**
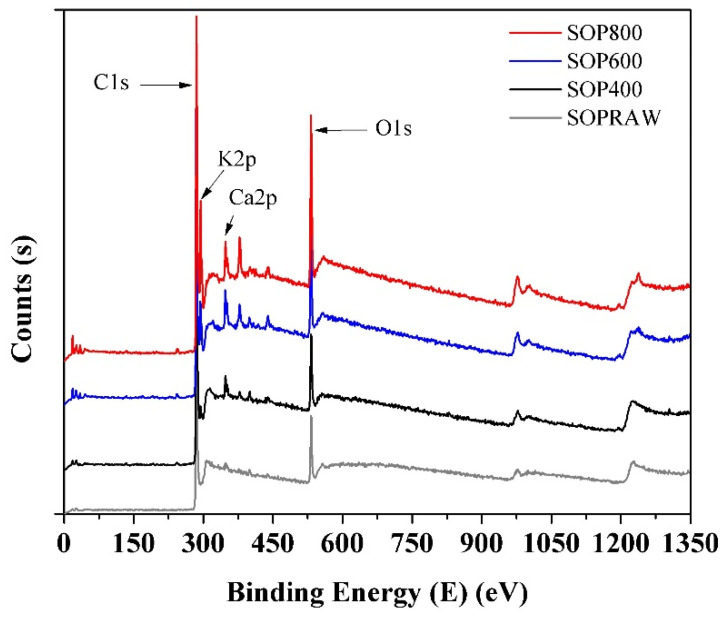
XPS survey spectra of SOPRAW and biochars.

Figure 8 shows the survey spectra of the SOPRAW and biochars, and the binding energies of 285, 293.08, 347.08, and 532 eV are attributed to C1s, K2p, Ca2p, and O1s, respectively. The obtained results show, firstly, that the main elements in the composition of biochars are carbon and oxygen, where the presence of a higher carbon content is observed as the pyrolysis temperature increases. Particularly, the peak of Ca2p begins to appear in SOPRAW and gradually increases with the pyrolysis temperature. In a similar way, the peak of K2p starts to appear in SOP400, which gradually increases and is more evident in SOP800. Both peaks are related to the presence of inorganic materials in the raw material and in the pyrolysis process and can be related to the ashes derived from the same process [44].

### 3.9. UV−VIS−NIR Optical Properties

**Figure 9:**
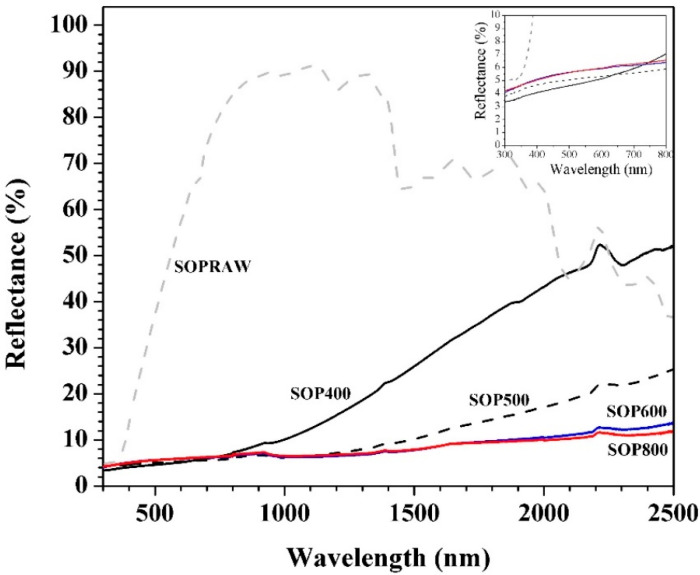
UV−VIS−NIR diffuse reflectance of SOPRAW and biochars.

Figure 9 shows the diffuse reflectance spectra of the biochars for the 300–2500 nm wavelength range. The diffuse reflectance spectra of SOPRAW shows a spectra with bands that can be related to the characteristic functional groups of lignocellulosic materials [52] where the changes occur after a pyrolysis process, and can be related with the decomposition of SOPRAW, as revealed in the FTIR analysis. It is also possible to appreciate that SOPRAW is a material that exhibits much higher diffuse reflectance values compared with those shown by the different biochars obtained, where a significant reduction of diffuse reflectance was achieved by pyrolyzing SOPRAW at 400 °C. The above indicates that the pyrolysis process favors the reduction of diffuse reflectance by means of the carbonization of the raw material. The main differences in the biochars are observed in the NIR interval, where SOP400 exhibited a higher diffuse reflectance than SOP600 and SOP800. In addition to this, a biochar at a pyrolysis temperature of 500 °C was obtained, where its diffuse reflectance behavior was also shown. It can be appreciated that in the NIR interval, SOP400 exhibits a diffuse reflectance from 7% to 52%, whereas SOP500, SOP600, and SOP800 show intervals of 6.2–25.5%, 6.6–13.8%, and 6.8-11.8% for their diffuse reflectance, respectively. Therefore, a decrease of diffuse reflectance is achieved by the increase in pyrolysis temperature, observing a more significant reduction when the pyrolysis temperature increases to 500–600 °C. Furthermore, a modest reduction on diffuse reflectance is achieved for a pyrolysis temperature of 600–800 °C; this can be attributed to the degree of decomposition of raw materials for each pyrolysis temperature. For SOP400, a partial decomposition is achieved, which is consistent with its physicochemical characterization, whereas for SOP600, a more thermal stable biochar is obtained because of decomposition reactions such as dehydration and deoxygenation, which produced a biochar with a higher carbon content with a higher graphitic degree and higher structural order, as the Raman analysis revealed. On the other hand, a modest reduction of diffuse reflectance was achieved between SOP600 and SOP800, despite SOP800 being a more ordered biochar with a higher graphitic degree than SOP600. This is consistent with the presence of the sp^2^−sp^3^ domains exhibited by the biochars in the Raman analysis. As mentioned, sp^2^−sp^3^ carbon allotropes exhibit attractive light absorption coefficients attributed to the sp^2^ domain of the graphitic structure [51], which are absent in SOPRAW. However, despite there being a reduction in diffuse reflectance as the pyrolysis temperature increases, and the biochar has a higher structural order; it seems that the effect of the sp_2_ domain starts to be limited when the temperature rises above 600 °C. Finally, the diffuse reflectance of the UV−VIS range is also shown, where diffuse reflectance showed little difference between biochars.

It is known that for efficient photothermal conversion, a solar absorber material must show a low reflectance at a UV−VIS−NIR interval. As was observed, the higher the pyrolysis temperature, the lower the diffuse reflectance. Then, by means of a pyrolysis temperature, it is possible to control this optical property. 

The diffuse reflectance behavior observed is similar to that found by Yang and Sheng, who used VIS−NIR spectroscopy to discriminate the biochar feedstock types and pyrolysis temperature, where diffuse reflectance in the VIS−NIR increases the wavelength by up to 10–30% for the reflectance values of the biochars based on wood and cotton stalk; where diffuse reflectance decreased with the pyrolysis and temperature, the lowest diffuse reflectance values were those exhibited by the highest pyrolysis temperatures [53]. Table 4 shows the solar absorption percentages in the UV−VIS−NIR interval of SOPRAW, SOP600, and SOP800, which were obtained from reflectance values and using the standard air 1.5 global (AM1.5G) solar spectrum [54]. The absorption values of the biochars from the literature are also shown. From Table 4, it can be appreciated that SOPRAW is the material with the lowest absorption values, whereas SOP600 and SOP800 exhibit the same absorption value, which is much higher than those exhibited by SOPRAW. On the other hand, by comparing the absorption percentages of SOP600 and SOP800 with the biochars from the literature as photothermal materials, it is shown that the biochars obtained from *Citrus aurantium* orange peel exhibited a potential to be used as a pigment for solar absorber coatings in solar-thermal applications.

## 4. Conclusions

The synthesis, physicochemical, and optical characterization of new biochars obtained from *Citrus aurantium* orange peel at three different temperatures was performed. The physicochemical characterization revealed that biochars with different resulting characteristics were produced. The carbonization yield was negatively affected by the pyrolysis temperature, whereas the carbon content and aromaticity improved with the increase of pyrolysis temperature, as revealed by the elemental analysis. In addition to this, the hydrophilicity index and thermal analysis showed that SOP600 and SOP800 exhibited a higher thermal stability than the SOP400 biochar, because only a partial decomposition of the raw material was achieved at 400 °C, as the thermal analysis and FTIR spectroscopy showed. On the other hand, the SEM micrographs showed important morphology changes in the raw material after the pyrolysis process. A morphology with cavities and the presence of small particles was more evident as the temperature increased, indicating a major decomposition degree, and then a higher carbonization was achieved. This is consistent with the DRX analysis, where the peaks corresponding to the planes of the graphitic structures were more evident. Moreover, the Raman analysis revealed an increase in the structural order of the biochars with a pyrolysis temperature, then SOP800 was the most ordered, which indicates that high pyrolysis temperatures favor the obtention of high ordered carbonaceous materials. Finally, it was observed that the increase of pyrolysis temperatures reduced the diffuse reflectance in the biochars. The main reduction on this optical property was produced from 400 °C to 600 °C, whereas only a modest reduction was achieved from 600 °C to 800 °C, where the SOP600 and SOP800 biochars exhibited a diffuse reflectance interval from approximately 6 to 14%, which indicates that pyrolysis temperature is an important condition to modify this property, which can be related to the degree of decomposition of the raw material and the presence of sp^2^−sp^3^ bonds. In addition to this, the biochars exhibited high solar light absorption values similar to those exhibited by the photothermal biochars in the literature. The resulting morphology, thermal stability, and presence of sp^2^−sp^3^ bonds, as well as optical properties such as diffuse reflectance and solar light absorption values exhibited by SOP600 and SOP800, suggest that the biochars obtained in this work showed very attractive physicochemical and optical properties to be used as a potential light absorbing material in solar absorber coatings, with the advantages of being low cost and environmentally friendly, which favors the utilization of agro-residues as an alternative to reduce the negative socioeconomic and environmental impacts caused by food and agro-waste.

## Figures and Tables

**Table 1:** Carbonization yield of the pyrolysis process of each temperature evaluated.

Sample	Pyrolysis Temperature (°C)	CY (%)
SOP400	400	36.35 ± 0.47
SOP600	600	28.83 ± 0.11
SOP800	800	27.14 ± 0.09

**Table 2:** Analysis of elemental composition (wt. % db) of SOPRAW and biochars.

Sample	% C	% H	% N	% S	% O *	H/C	O/C
SOPRAW	47.82 ± 1.48	5.99 ± 0.06	0	0	46.09 ± 1.54	0.125 ± 0.003	0.963 ± 0.062
SOP400	54.6 ± 7.44	3.51 ± 0.48	0	0	41.89 ± 7.92	0.064 ± 0.000	0.784 ± 0.252
SOP600	60.59 ± 0.99	1.65 ± 0.05	0	0	37.77 ± 1.04	0.027 ± 0.000	0.624 ± 0.027
SOP800	72.26 ± 0.62	0.88 ± 0.01	0	0	26.86 ± 0.64	0.012 ± 0.000	0.372 ± 0.012

* Obtained by difference (100-C-H-N-S); db—dry basis.

**Table 3:** Disorder degree of SOPRAW and biochars SOP400, SOP600, and SOP800.

Sample	I_D_/I_G_	I_D_/(I_D_ + I_G_)
SOP400	0.581	0.367
SOP600	0.700	0.411
SOP800	0.926	0.481

**Table 4:** Solar absorption percentages in the UV−VIS−NIR interval of SOPRAW and biochars.

Sample	Absorption (%)
SOPRAW	48.7 ± 18.7
SOP600	94.1 ± 0.5
SOP800	94.1 ± 0.5
Biochar from loofah sponge pyrolyzed at 800 °C [54]	84
Biochar from daikon pyrolyzed at 750 °C [55]	90–95

## Data Availability

No data availability.
